# Agmatine suppresses glycolysis via the PI3K/Akt/mTOR/HIF‐1α signaling pathway and improves mitochondrial function in microglia exposed to lipopolysaccharide

**DOI:** 10.1002/biof.2149

**Published:** 2025-01-30

**Authors:** Katarina Milosevic, Ana Milosevic, Ivana Stevanovic, Anica Zivkovic, Danijela Laketa, Marija M. Janjic, Ivana Bjelobaba, Irena Lavrnja, Danijela Savic

**Affiliations:** ^1^ Department of Neurobiology Institute for Biological Research “Sinisa Stankovic”—National Institute of Republic of Serbia, University of Belgrade Belgrade Serbia; ^2^ Medical Faculty of the Military Medical Academy University of Defense in Belgrade Belgrade Serbia; ^3^ Department for General Physiology and Biophysics Faculty of Biology, University of Belgrade Belgrade Serbia

**Keywords:** agmatine, ATP, glycolysis, HIF‐1α, microglia, PI3K/Akt/mTOR

## Abstract

Modulating metabolic pathways in activated microglia can alter their phenotype, which is relevant in uncontrolled neuroinflammation as a component of various neurodegenerative diseases. Here, we investigated how pretreatment with agmatine, an endogenous polyamine, affects metabolic changes in an in vitro model of neuroinflammation, a murine microglial BV‐2 cell line exposed to lipopolysaccharide (LPS). Hence, we analyzed gene expression using qPCR and protein levels using Western blot and ELISA. Microglial metabolic status was assessed by measuring lactate release and cellular ATP by enzymatic and luminescence spectrophotometry. Mitochondrial functionality was analyzed by fluorescent probes detecting mitochondrial membrane potential (mtMP) and superoxide production. Our findings suggest that kinase pathways associated with hypoxia‐inducible factor‐1α (HIF‐1α) regulate energy metabolism in pro‐inflammatory activated microglia. We have shown that LPS induces HIF‐1α and genes for glucose transporter and glycolytic rate, increases lactate production and causes mitochondrial dysfunction, suggesting a metabolic shift towards glycolysis. Agmatine inhibits the PI3K/Akt pathway and negatively regulates mammalian target of rapamycin (mTOR) phosphorylation and HIF‐1α levels, reducing lactate and tumor necrosis factor (TNF) production, which is supported by pharmacological blockade of PI3K. Pretreatment with agmatine also rescues mitochondrial function by counteracting the LPS‐induced decline in mtMP and increase in mitochondrial superoxide, resulting in an anti‐apoptotic effect. Agmatine alone increases intracellular ATP levels and maintains this effect even under pro‐inflammatory conditions. Our study emphasizes the ability of agmatine to engage in metabolic reprogramming of pro‐inflammatory microglia through increased ATP production and modulation of signaling pathway involved in promoting glycolysis and cytokine release.

## Graphical abstract

Agmatine, an endogenous polyamine, changes metabolism of pro‐inflammatory microglia via the PI3K/Akt/mTOR/HIF‐1α signaling pathway. Created with BioRender.com.

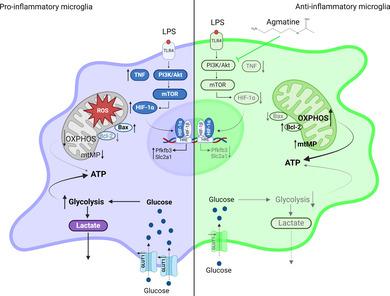

**Abbreviations**
Agm: agmatine
BSA: bovine serum albumin
CCCP: carbonyl cyanide 3‐chlorophenylhydrazone
CNS: central nervous system
ELISA: enzyme‐linked immunosorbent assay
FBS: fetal bovine serum
GLUT1: glucose transporter 1
HIF‐1α: hypoxia‐inducible factor‐1α
iNOS: inducible nitric oxide synthase
LDH: lactate dehydrogenase
LPS: lipopolysaccharide
LY: LY294002
MTG: MitoTracker Green
mtMP: mitochondrial membrane potential
mTOR: mammalian target of rapamycin
NF‐κB: nuclear factor kappa‐B
NO: nitric oxide
NOS: nitric oxide synthase
OXPHOS: oxidative phosphorylation
PD: Parkinson's disease
PFA: paraformaldehyde
PFKFB3: 6‐phosphofructo‐2‐kinase/fructose‐2,6‐bisphosphatase 3
qPCR: quantitative real‐time PCR
RNS: reactive nitrogen species
ROS: reactive oxygen species
RTCA: real‐time cell analysis
TLR4: Toll‐like receptor 4
TMRE: tetramethylrhodamine ethyl ester
TNF: tumor necrosis factor
Wort: wortmannin

## 1 INTRODUCTION

The central nervous system (CNS) is equipped with innate immune cells, the microglia, which play a crucial role in maintaining balance, monitoring threats, and providing defense. If harmful molecules are detected, microglia undergo various functional changes leading to a transition towards a pro‐ or anti‐inflammatory state. These changes involve dynamic modifications in the cell's structure, generating reactive oxygen and nitrogen species (ROS, RNS, respectively), releasing inflammatory agents, engulfing foreign particles, and moving towards the site of inflammation or injury. This rapid reaction is vital for eliminating infections and cleaning and repairing damaged tissue in the brain. Most neurodegenerative diseases are associated with chronic neuroinflammation, in which control over microglia activation has been lost.^1^ In these cases, microglia typically become overactived for prolonged periods, contributing significantly to disease progression and exacerbating CNS damage rather than protecting it. The importance of influencing microglial activation and polarization is underscored as a strategy for combating pathological neuroinflammation.

The coordination between microglia's activation and energy metabolism is crucial to their defense response.^2^ Microglia's metabolic profile undergoes significant changes during an inflammatory response and adjusts to the energy needs of the activated state.^3^ These changes result in a decrease in oxidative phosphorylation (OXPHOS) and ATP synthesis in the mitochondria. Concomitantly, the cells use more glucose and produce and excrete more lactate, regardless of the presence of oxygen. The metabolic shift during the immune response, favoring aerobic glycolysis and lactate production, is similar to the Warburg effect, first observed in cancer cells.^4^ In contrast to the Warburg effect in inflammation and tumors, astrocytes in the brain use the Warburg principle of lactate production during neuronal stimulation. The lactates are then transported to the neurons, which use them as energy carriers and signaling molecules that influence plasticity and excitability.^5^, ^6^ Increasing glycolysis in an activated immune cell produces ATP at a higher rate than OXPHOS, supports the supply of metabolic precursors repurposed to promote the production of inflammatory mediators and enables significant cytoskeletal rearrangements necessary for immune cell migration.^7^, ^8^ Enhanced glycolysis is the universal response of immune cells to various pathogen‐associated molecular patterns, including LPS‐induced Toll‐like receptor 4 (TLR4) activation.^9^ Indeed, measurements of cellular respiratory control show that LPS induces glycolytic shift in primary microglia and the BV‐2 cell line.^10^, ^11^, ^12^ Interestingly, anti‐inflammatory microglial activation probably requires an increase in OXPHOS.^13^ These findings highlight the potential therapeutic implications of modulating microglial metabolism to elicit specific activation phenotypes.^10^, ^13^, ^14^

Hypoxia‐independent activation of hypoxia‐inducible factor‐1α (HIF‐1α) was observed in the pro‐inflammatory activation of microglia,^3^, ^15^ where it plays a dual role: in regulating energy metabolism and inducing pro‐inflammatory mediators.^3^, ^14^, ^16^ Usually, when oxygen is present, the gene expression of HIF‐1α is high, but oxygen‐dependent prolyl hydroxylases rapidly mark the HIF‐1α protein for proteasomal degradation. In addition, HIF‐1α may be regulated at the transcriptional or translational level by signaling pathways that activate inflammatory responses in microglia.^12^, ^15^, ^16^ For example, nuclear factor kappa‐B (NF‐κB) and specificity protein 1 promote the transcription of HIF‐1α.^15^, ^17^ Another signaling pathway, PI3K/Akt, whose activation has both anti‐inflammatory and pro‐inflammatory effects, can indirectly stimulate HIF‐1α protein synthesis by targeting the mammalian target of rapamycin (mTOR).^12^, ^16^ Once HIF‐1α accumulates and translocates to the nucleus, it assumes the role of a transcription factor, promoting the expression of genes encoding glucose transporters or glycolytic enzymes such as hexokinases, pyruvate dehydrogenase kinase‐1 and 6‐phosphofructo‐2‐kinase/fructose‐2,6‐bisphosphatase 3 (PFKFB3), resulting in increased glycolysis.^18^

Dependence on aerobic glycolysis in LPS‐challenged microglia is associated with reduced mitochondrial function and structural changes in these organelles, recorded as excessive mitochondrial fragmentation.^10^ LPS also causes alterations in the expression and levels of certain members of the mitochondrial uncoupling protein family, affecting mitochondrial membrane potential (mtMP).^19^ As the protons are not utilized as a driving force for ATP synthesis an increase in mtMP was observed during the LPS‐induced reduction of OXPHOS in macrophages and microglia.^10^, ^20^ The purpose of all these mitochondrial changes during the switch to glycolysis is to redirect the electron transport chain from ATP synthesis to ROS production, as demonstrated in macrophages.^20^ Moreover, an increase in mitochondrial ROS is also a characteristic of pro‐inflammatory microglia^10^, ^11^, ^19^ and is essential for regulating key pro‐inflammatory signaling pathways such as mitogen‐activated protein kinase and NF‐kB.^21^

Agmatine, an endogenously produced amino compound, acts as a multifunctional neuromodulator in the mammalian brain, affecting neurotransmission, inhibition of nitric oxide synthase, and polyamine regulation, thus supporting cellular homeostasis and neural health. Although its exact endogenous role is not yet fully understood, agmatine fulfills several neurotransmitter criteria, including specific synthesis, storage and regulated release upon neuronal depolarization.^22^, ^23^ Agmatine is formed as part of the arginine metabolism through arginine decarboxylase (ADC) activity and degraded by agmatinase. ADC, found in neurons and astrocytes, has a low activity in mammalian tissues. Therefore, only a tiny fraction of endogenous agmatine arises from cellular enzymatic de novo synthesis, while the primary source is probably food.^24^, ^25^ The enzymes ADC and agmatinase, along with the agmatine‐binding imidazoline receptor I2, are localized in the mitochondria.^26^, ^27^, ^28^ Agmatine's ability to enter the mitochondrial matrix of liver and brain cells via specific transporters further underscores the importance of its mitochondrial association.^29^, ^30^

Agmatine is known for its multiple neuroprotective effects in various neuropathologies, from ischemia, traumatic CNS injuries and epilepsy to neurodegenerative diseases and neuropsychiatric disorders, as shown in preclinical studies and in vitro systems.^25^ Neuroinflammation often significantly contributes to the pathology of these diseases and is therefore considered a possible therapeutic target. Three recent studies have identified molecular targets of agmatine in BV‐2 microglia stimulated with LPS that contribute to the reduction of microglial pro‐inflammatory properties and polarization into an anti‐inflammatory phenotype.^31^, ^32^, ^33^

Considering the metabolic switch of immune cells to glycolysis in response to pro‐inflammatory stimuli and the anti‐inflammatory effect of agmatine in BV‐2 cells, we hypothesized that agmatine might contribute to the reduction of inflammation by suppressing glycolysis. To test this, we challenged the BV‐2 microglial cell line with LPS and analyzed the timeline of metabolic changes to understand if and when agmatine treatment affects glycolysis. We focussed on determining the levels of HIF‐1α and its regulators and effectors, as well as intracellular ATP and extracellular lactate concentrations. Since the enzymatic machinery of endogenous agmatine is found in the mitochondria and agmatine can enter these organelles, we also investigated whether it improves LPS‐induced mitochondrial dysfunction. To this end, we investigated mtMP, mitochondrial superoxide production and mitochondrial apoptotic signaling molecules. The interdependence of the metabolic and activating microglial phenotypes emphasizes the importance of identifying molecular targets and mechanisms that can effectively influence both aspects. The optimal manipulation of these targets is particularly important in a complex CNS environment where controlling neuroinflammation is essential.

## 2 EXPERIMENTAL PROCEDURES

### 2.1 Cell culture

BV‐2 mouse microglial cell line was purchased from Elabscience Biotechnology Inc. (Houston, TX, USA, Cat. No. EP‐CL‐0493) and used in all experiments. For each independent experiment, we thawed different culture batches. We conducted three to five independent experiments (3 ≤ *n* ≤ 5), always completing one experiment before starting the next to ensure their independence. Each ‘*n*’ represents an average of three to five replicates per group. Cells were grown in RPMI 1640 medium containing 10% heat‐inactivated fetal bovine serum (FBS), 100 U/mL penicillin, and 100 μg/mL streptomycin (all from Gibco, Thermo Fisher Scientific, Waltham, MA, USA) at 37°C and 5% CO_2_ in a humidified environment. After reaching 80%–90% confluence, the cells were passaged and seeded for further analysis. After 21 h, the medium with 10% FBS was replaced with a serum‐reduced medium containing 1% bovine serum albumin (BSA, Sigma‐Aldrich, Munich, Germany), and the cells were adapted for 3 h. Afterwards, the cells were pretreated with 100 μM agmatine sulfate salt (Agm, Sigma‐Aldrich, St. Louis, MO, USA) for 30 min and then stimulated with 1 μg/mL Lipopolysaccharide (LPS from *Escherichia coli* serotype 026:B6; Sigma‐Aldrich Labware, Munich, Germany) for various incubation times, depending on the experimental objective. We defined the following experimental groups: untreated control cells (Ctrl), Agm‐treated cells (Agm), LPS‐stimulated cells (LPS), and LPS‐stimulated cells pretreated with Agm (LPSAgm). A set of experiments was conducted using known PI3K inhibitors, wortmannin (Wort, EMD Chemicals, San Diego, CA, USA; final concentration 100 nM) and LY294002 (LY, Cell Signaling Technology, Danvers, MA, USA; final concentration 10 μM), administered 1 h before LPS stimulation.

### 2.2 Real‐time cell analyzing of BV‐2 microglia

The response of BV‐2 cells to Agm and LPS exposure was determined by continuous cell monitoring in real‐time using the label‐free xCELLigence Real‐Time Cell Analysis biosensor technology (RTCA SP, Agilent, Santa Clara, CA, USA). The device operates a 96‐well electronic microplate (E‐Plate 96) with gold microelectrodes on the bottom of the wells that allow electronic flow in an electrically conductive solution such as the culture medium. This system measures the electronic flux impedance created by the adhesive cells' interaction with the electrode‐solution interface, expressed as the Unitless parameter Cell Index, which reflects changes in cellular/molecular events such as viability, proliferation, morphology, and adhesion. BV‐2 microglia were seeded in 96‐well gold electronic microplate (E‐plate) at a density of 20,000 cells/well and next day, after the medium replacement procedure, pretreated with Agm for 30 min and then stimulated with LPS for 24 h. The Cell Index was logged every 5 min throughout the entire experiment, and time points at 3 h intervals following LPS administration were selected for analysis. To avoid the influence of variations in initial cell seeding density or attachment, the Cell Index was normalized, providing a uniform starting point for all groups. The normalized Cell Index (nCell Index) was determined by dividing the Cell Index at each time point by the Cell Index at the time of LPS application. Data are from four separate experiments, performed at least in quintuplicate for each experimental group and are expressed as the mean of the nCell Index ± SEM.

### 2.3 Measurement of intracellular ATP concentration

BV‐2 cells were grown in 6‐well plates (300,000 cells/well) and subjected to Agm treatment followed by LPS stimulation for 3, 6, and 24 h. Cellular ATP was extracted with boiling water for the luciferin‐luciferase assay of ATP as previously described.^34^ ATP concentration was determined using bioluminescent ATP kits from Sigma Aldrich (St. Louis, MO, USA) according to the manufacturer's instructions and luminescence was measured using a luminometer (CHAMELEON™V, Hidex, Turku, Finland). ATP content was normalized to the total cellular protein concentration. Results are from three to five independent experiments (each group was performed in triplicate) and are expressed as mean ATP concentration (nmol/mg protein) ± SEM.

### 2.4 Gene expression analysis

Cells were seeded in 6‐well plates (600,000 cells/well) to extract total RNA 2, 4, and 6 h after treatment with the RNeasy mini kit (QIAGEN, Hilden, Germany). RNA concentration and purity were determined using a Nanophotometer® N60 (IMPLEN, Munich, Germany), and 1 μg of RNA was used for reverse transcription with the High Capacity cDNA Reverse Transcription Kit (Applied Biosystems by Thermo Fisher Scientific, Waltham, MA, USA). The cDNA samples were then analyzed for gene expression by quantitative real‐time PCR (qPCR) on the QuantStudio™ 3 Real‐Time PCR System (Applied Biosystems by Thermo Fisher Scientific) using SYBR™ Green reagent (Applied Biosystems by Thermo Fisher Scientific). The primers of the target genes were designed as follows: *Hif1a* (F: 5′‐TCAAGTCAGCAACGTGGAAG; R: 5′‐TATCGAGGCTGTGTCGACTG; accession number: NM_010431.2), *Slc2a1* (F: 5′‐GAGTGACGATCTGAGCTACGG; R: 5′‐CTCCCACAGCCAACATGAGG; accession number: NM_011400.3) and *Pfkfb3* (F: 5′‐GTCGCCGAATACAGCTACGA; R: 5′‐GAGCCCCACCATCACAATCA; accession number: NM_001177752.1). The expression levels of the target genes were calculated using the comparative 2^−∆Ct^ method, using *Actb* as the housekeeping gene (F: 5′‐GGGCTATGCTCTCCCTCAC; R: 5′‐GATGTCACGCACGATTTCC; accession number: NM_007393.5). The results are presented as the mean of the expression ratios between the target genes and *Actb* ± SEM from three or four independent experiments, with triplicate per group.

### 2.5 Western blot analysis

Samples were prepared from cells cultured in 6‐well plates (600,000 cells/well), lysed with ice‐cold Triton X‐100 buffer saline mixed with protease and phosphatase inhibitors (Thermo Fisher Scientific, Waltham, MA, USA), and pooled from five replicates of each group. 15 μg (for p‐Akt, Akt, p‐PI3K, and PI3K) or 20 μg (for Bax, Bcl‐2, HIF‐1α, p‐mTOR, mTOR, and GLUT1) of proteins per group were separated during electrophoresis on an 8% (p‐mTOR and mTOR), 10% (HIF‐1α, p‐PI3K, PI3K, and GLUT1) or 12% SDS‐polyacrylamide gel (Bax, Bcl‐2, p‐Akt, and Akt). All subsequent steps were performed as previously described,^31^ with the modification of p‐mTOR and mTOR transfer, which lasted 90 min at 100 V. All antibodies used in this study are listed in Table 1. ImageQuant 5.2 software was used for densitometric analysis of the protein bands, and the optical density of the target protein band was normalized to the optical density of the β‐actin of the same lane. The data shown are from four independent determinations expressed as mean relative to control (fold change) ± SEM.

**TABLE 1:** List of antibodies used for Western blot (WB) and immunofluorescence (IF) microscopy.

Antibody	Host and clonality	Dilution and application	Company and catalogue code
Bax	Rabbit, polyclonal	1:500, WB	Santa Cruz, sc‐493
Bcl‐2	Rabbit, polyclonal	1:1000, WB	Cell Signaling, #2876S
HIF‐1α	Rabbit, polyclonal	1:1000, WB; 1:200, IF	Cell Signaling, #3716S
Phospho‐PI3K	Rabbit, polyclonal	1:1000, WB	Cell Signaling, #4228
PI3K	Rabbit, monoclonal	1:1000, WB	Cell Signaling, #4257
Phospho‐Akt	Rabbit, monoclonal	1:1000, WB	Cell Signaling, #2965
Akt	Rabbit, polyclonal	1:1000, WB	Cell Signaling, #9272
Phospho‐mTOR	Rabbit, polyclonal	1:1000, WB	Cell Signaling, #2971
mTOR	Rabbit, polyclonal	1:1000, WB	Cell Signaling, #2972
GLUT1	Rabbit, polyclonal	1:4000, WB	Abcam, ab652
β‐Actin	Mouse, monoclonal	1:5000, WB	Sigma Aldrich, A5316
Anti‐rabbit IgG AlexaFluor 555	Donkey	1:250, IF	Invitrogen, A31572
Anti‐rabbit IgG‐HRP	Goat	1:5000, WB	Santa Cruz, sc‐2004
Anti‐rabbit IgG‐HRP	Donkey	1:5000, WB	Santa Cruz, sc‐2313
Anti‐mouse IgG‐HRP	Donkey	1:5000, WB	Santa Cruz, sc‐2314

### 2.6 Immunofluorescence staining

Fluorescence immunocytochemistry was performed on cells seeded on PLL‐coated Ø25 glass coverslips (50,000 cells) and fixed in cold 4% paraformaldehyde (PFA) for 20 min 3 h after LPS stimulation. The cells were first permeabilized with ice‐cold methanol for 5 min in the freezer and then with 0.25% Triton X‐100 for 15 min at room temperature. After rinsing with PBS, the cells were blocked with 3% BSA and incubated overnight at 4°C with the primary anti‐HIF‐1α antibody. The next day, the cells were rinsed and incubated for 1 h in the dark with the secondary antibody (Table 1). The cell nuclei were stained with Hoechst 33258 (Sigma‐Aldrich, St. Louis, MO, USA). The stained cells were visualized using fluorescence microscopy and the Zeiss Axiovert microscope (Zeiss, Jena, Germany). The images were taken with the same exposure time for all experimental groups.

### 2.7 Measurement of lactate production

Lactate concentration was determined by enzymatic spectrophotometric analysis based on lactate dehydrogenase (LDH) activity measurement, assessed by the amount of NADPH produced, which is directly proportional to the lactate concentration in the sample. The lactate concentration of the samples was calculated using a standard curve generated from lactic acid (Sigma‐Aldrich, St. Louis, MO, USA). For this purpose, the culture medium was collected from 6‐well plates with 600,000 cells per well 3, 6, and 24 h after LPS application. The samples were deproteinized with ice‐cold 8% (w/v) aqueous perchloric acid (Merck Millipore, Darmstadt, Germany) and centrifuged at 1500 × g at 4°C for 10 min. 50 μL of the prepared supernatants or standards were incubated with a reaction mixture containing glycine buffer (0.6 M glycine and 0.5 M hydrazine, pH 9.2), 2.5 mM β‐NAD, and 25 U LDH (all from Sigma‐Aldrich, St. Louis, MO, USA) in a final volume of 1.5 mL, for 15 min at 37°C. The absorbance of the samples was measured at 340 nm using a UV–visible spectrophotometer (Shimadzu UV‐1900i, Kyoto, Japan). Results are normalized to protein concentration, obtained from three or four independent experiments for each time point (triplicates per group), and expressed as mean lactate concentration (mM/mg) ± SEM.

### 2.8 Enzyme‐linked immunosorbent assay (ELISA)

Cells were incubated with Wort and LY for 1 h or pretreated with Agm 30 min before LPS stimulation. After 24 h, supernatants were collected and adequately diluted for analysis. Tumor necrosis factor (TNF) production in the samples was measured by ELISA as described in our previous study.^31^ A standard curve was generated using a recombinant mouse TNF (Cat. no. ab212073, Abcam, Cambridge, UK), and the concentration of TNF in the samples was determined using a 5‐PL logistic curve and normalized to protein content. Data are presented as mean ± SEM from three independent experiments, performed in duplicates per group.

### 2.9 Determination of mitochondrial superoxide

The production of mitochondrial superoxide in BV‐2 microglia was determined using the indicator MitoSOX™ Red (Invitrogen by Thermo Fisher Scientific, Waltham, MA, USA). MitoTracker Green (MTG, Invitrogen by Thermo Fisher Scientific, Waltham, MA, USA) determined mitochondrial content in living cells. The method was conducted in a black 96‐well plate with a clear bottom. After Agm pretreatment and LPS stimulation for 3, 6 (40,000 cells/well), and 24 h (15,000 cells/well), 200 nM MTG was applied for 20 min. The cells were then rinsed with Hanks' Balanced Salt Solution (Biowest, Nuaillé, France) and incubated with 5 μM MitoSOX at 37°C for 30 min. After washing the cells, the fluorescence signal was detected at Ex/Em: 480/530 for MTG and Ex/Em: 510/580 for MitoSOX using the microplate reader (Synergy H1M). Immediately after analysis on the microplate reader, cells were visualized with the ZOE Fluorescent Cell Imager (Bio‐Rad Laboratories, Inc., Hercules, CA, USA) by imaging fluorescence at the same exposure for all groups. The results are expressed as the mean of the MitoSox and MTG ratio ± SEM of four independent experiments performed in quintuplicate per group.

### 2.10 Mitochondrial membrane potential detection

Changes in mtMP were detected using a fluorometric assay based on the staining cells with tetramethylrhodamine ethyl ester (TMRE, Invitrogen by Thermo Fisher Scientific, Waltham, MA, USA). BV‐2 cells were seeded in a black 96‐well plate with a clear bottom and treated with Agm and LPS for 3, 6 (40,000 cells/well), and 24 h (15,000 cells/well). In addition, cells treated with carbonyl cyanide 3‐chlorophenylhydrazone (CCCP, Sigma Aldrich, St. Louis, MO, USA), known to dissipate the mtMP, were a positive control for mitochondrial membrane depolarisation. 10 μM CCCP was administered 5 min before incubation of cells with 200 nM MTG and 200 nM TMRE for 20 min at 37°C. The cells were then washed, and fluorescence analysis of MTG at Ex/Em: 480/530 nm and TMRE at Ex/Em: 550/580 nm was performed on the microplate reader (Synergy H1M). Since MTG binds to mitochondrial proteins independent of mtMP, it indicates cellular mitochondrial mass. In addition, cell imaging with the ZOE Fluorescent Cell Imager (Bio‐Rad Laboratories) was performed immediately after measuring fluorescence intensity with the microplate reader, following the same procedure as for MitoSOX analysis. Results are expressed as the mean of the ratio between the relative fluorescence units of TMRE and MTG ± SEM from four independent experiments in quintuplicate per group.

### 2.11 Data analysis

All results were statistically processed using SPSS 20 software (IBM, Armonk, NY, USA) and GraphPad Prism 8.0.2 software (GraphPad Software Inc., La Jolla, CA, USA). Before selecting the statistical test for result analysis, the data were tested for normality using the Kolmogorov–Smirnov test and for equality of variances using Levene's test. When the data satisfied the assumptions of normal distribution and homogeneity of variance, a two‐way ANOVA followed by Tukey's post hoc test was used to analyze the means of multiple groups involving two factors (treatment and stimulation), while the data involving only stimulation as a factor underwent one‐way ANOVA followed by Dunnett's multiple comparisons test. The nonparametric Mann–Whitney test was used to compare two groups. The results are expressed as mean ± SEM, with the significance level denoted by *p < 0.05 compared to the control group and #*p* < 0.05 compared to the LPS group.

## 3 RESULTS

### 3.1 Real time monitoring of BV‐2 cells under stimulation and agmatine treatment

**FIGURE 1:**
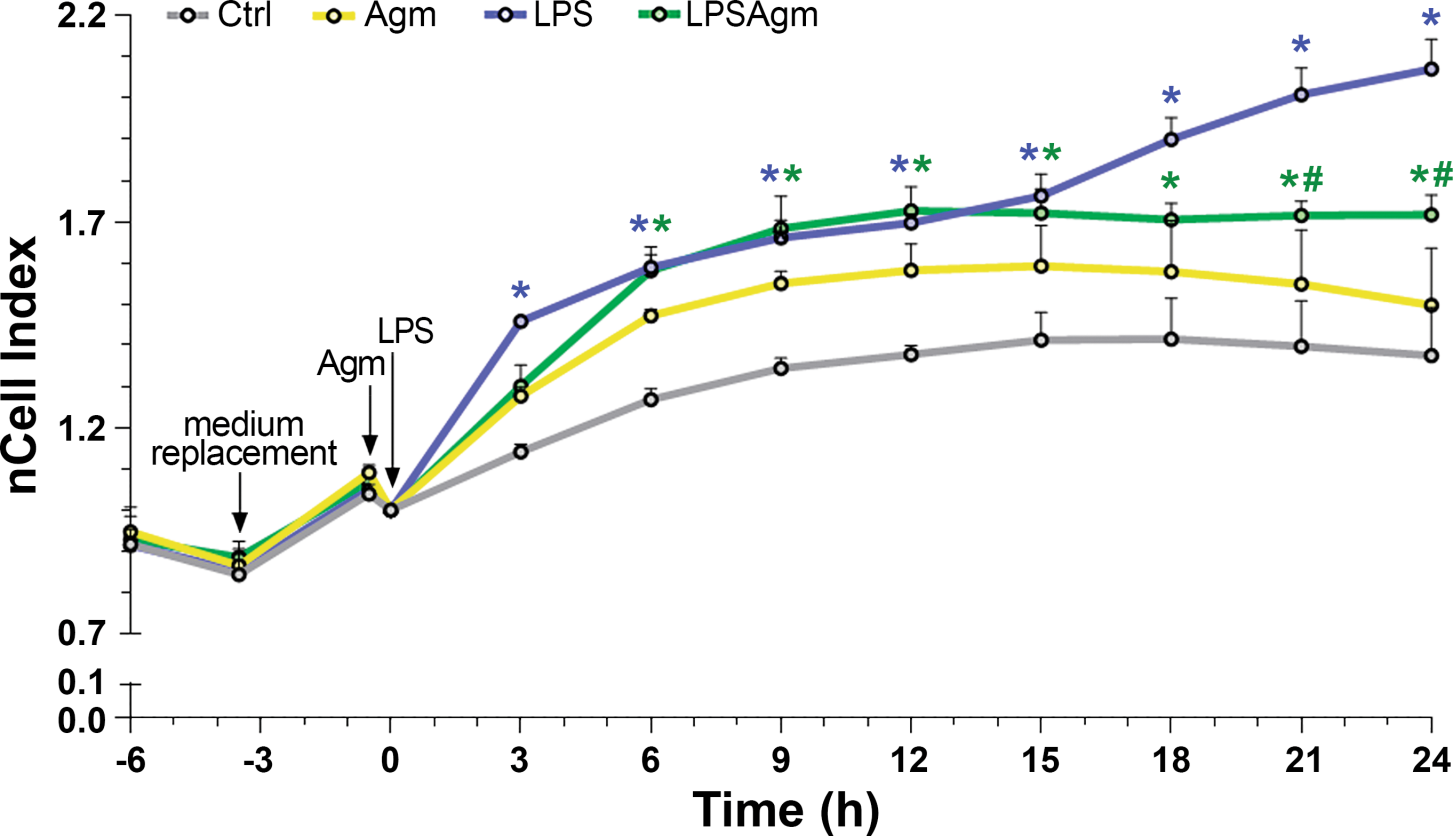
Real‐time analysis of agmatine effect on BV‐2 microglia cell status continuously over 24 h after LPS stimulation with the xCELLigence system. Cells were seeded in RPMI containing 10% FBS that was replaced after 21 h with serum‐reduced medium (RPMI containing 1% BSA). After 3 h of adaptation, cells were pretreated with 100 μM Agm for 30 min and then treated with 1 μg/mL LPS for 24 h. The graph represents the normalized Cell Index (nCell Index) of control cells (Ctrl, gray line), agmatine‐treated cells (Agm, yellow line), LPS‐stimulated cells (LPS, blue line), and cells pretreated with agmatine and then stimulated with LPS (LPSAgm, green line). Results are presented as mean nCell Index ± SEM from four independent experiments (*n* = 4). Results were analyzed using two‐way ANOVA followed by Tukey's post hoc test. Blue and green *p < 0.05 for the comparison of the LPS and LPSAgm with the control group, respectively; green #*p* < 0.05 compared with LPS group. In the timeline, the LPS addition time is set to zero.

The RTCA software was used to continuously monitor the cellular status of BV‐2 microglia in response to Agm treatment and LPS stimulation in real time for 24 h (Figure 1). LPS significantly increased the nCell Index of microglia after 3 h of administration compared to the control. The nCell Index continued to increase until the 24th hour. Agm alone did not affect the nCell Index. Under inflammatory conditions, an increase in the nCell Index of Agm‐treated cells compared to the control was observed later than in LPS‐treated cells – after 6th hour and remained at the same level until the end of the observation period. Agm treatment also reduced the changes induced by LPS from the 21st to the 24th hour. Noteworthy, cell biomass, estimated using crystal violet assay, was not affected by either LPS or Agm administration (Figure S1). However, the MTT assay showed increased metabolic activity in the stimulated cells, independent of Agm treatment (Figure S1). These results indicate that the differences observed in the nCell Index of the stimulated microglia compared to the control cells are not consequences of interrupted viability. However, our results show LPS‐induced cell enlargement, while Agm pretreatment leads to a reduction in cell size of LPS‐stimulated BV‐2 cells (Figure S2), which is consistent with the observed changes in nCell Index. To further investigate time‐dependent metabolic changes in microglia after LPS stimulation, we carefully selected three time points for analysis: 3, 6, and 24 h.

### 3.2 Agmatine increases ATP production independent of the activation status of BV‐2 cells

**FIGURE 2:**
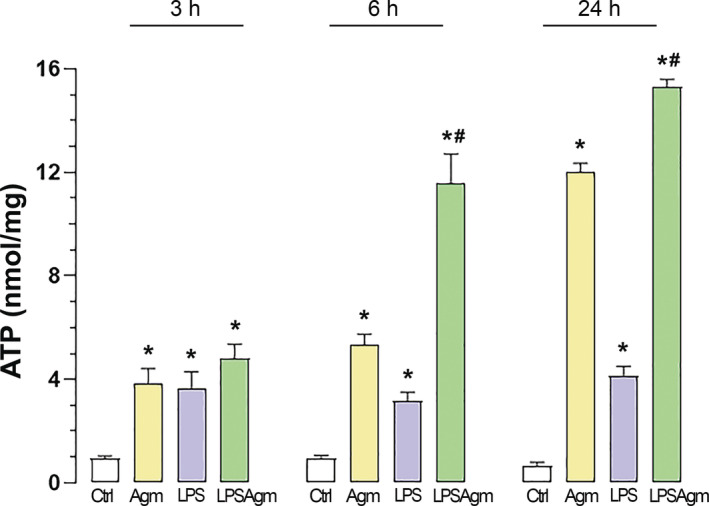
The effect of agmatine on intracellular ATP levels in BV‐2 microglia. Cells were assimilated for 3 h under serum‐reduced conditions before pretreatment with Agm (100 μM) for 30 min, followed by LPS stimulation (1 μg/mL) for 3, 6, and 24 h. Intracellular ATP concentrations were expressed in nmol per mg protein, representing the mean of three to five independent experiments ± SEM (3 ≤ *n* ≤ 5). Results were analyzed using a two‐way ANOVA, followed by Tukey's post hoc test for statistical significance: *p < 0.05 compared to the control group; #*p* < 0.05 compared to the LPS group.

Considering the metabolic changes in microglia caused by activation and the associated energy demand, we analyzed the effect of Agm pretreatment on intracellular ATP concentration 3, 6, and 24 h after stimulation by LPS (Figure 2). After (3 h) LPS stimulation, the energy status of the cells changes, as evidenced by an increase in intracellular ATP concentration compared to the control. These LPS‐induced changes persisted at 6 and 24 h upon stimulation. Agm significantly increased ATP concentrations after 3 h with a progressive trend over time in both control and LPS‐stimulated cells. These results suggest that both LPS and Agm affect ATP‐producing metabolic pathways, with Agm having a stronger influence on ATP production than LPS.

### 3.3 Agmatine attenuates LPS‐triggered upregulation of HIF‐1α

**FIGURE 3:**
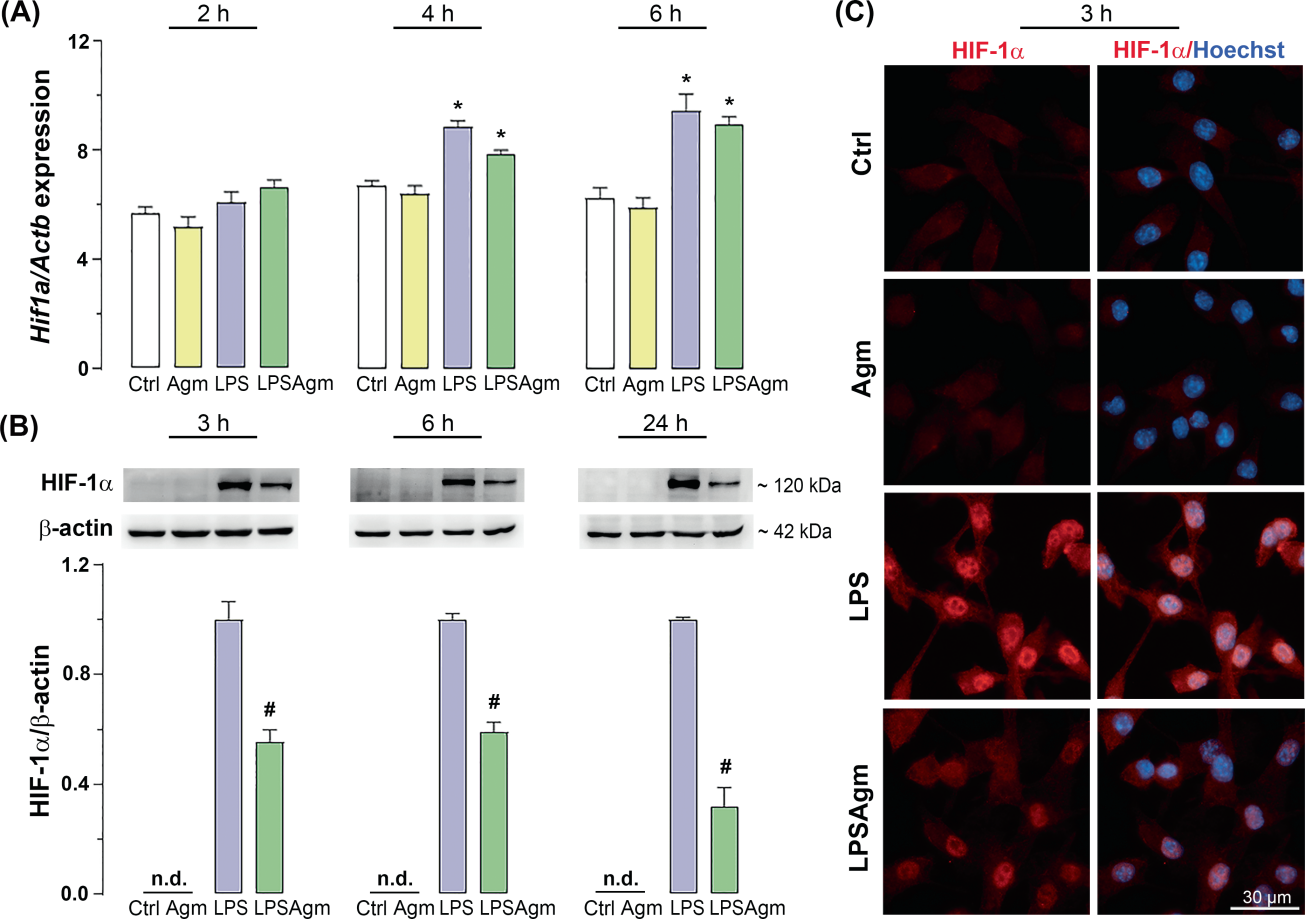
Agmatine effect on the gene expression and protein levels of HIF‐1α in activated microglia. BV‐2 cells underwent a 3‐h assimilation process in the serum‐reduced medium. They were pretreated with Agm (100 μM) for 30 min before stimulation with LPS (1 μg/mL) for the following time points: 2, 4, and 6 h for qPCR analysis; 3, 6, and 24 h for Western blot analysis; and 3 h for immunofluorescence analysis. (A) Gene expression of *Hif1a* relative to the expression of the *Actb* gene. Data are analyzed using a two‐way ANOVA, followed by Tukey's post hoc test and presented as mean ± SEM from three independent isolations (*n* = 3). (B) HIF‐1α protein levels relative to β‐actin expressed as LPS fold change ± SEM of four independent experiments for each time point (*n* = 4). The Mann–Whitney test was used to compare the mean values between two groups (LPS vs. LPSAgm), as HIF‐1α was not detectable (n.d.) in the Ctrl and Agm groups. Significance level: *p < 0.05 compared to the control group; #*p* < 0.05 compared to the LPS group. (C) Localisation of the HIF‐1α protein (red) in the cell nucleus (marked with Hoechst, blue) 3 h after administration of LPS. Scale bar = 30 μm.

Since the metabolic switch to glycolysis under inflammatory conditions is associated with a HIF‐1α‐dependent mechanism, we analyzed HIF‐1α gene expression 2, 4, and 6 h, and protein levels 3, 6, and 24 h after LPS administration (Figure 3). *Hif1a* was upregulated in LPS‐exposed cells after 4 and 6 h, while no significant changes were observed in control cells treated with Agm. Pretreatment of the stimulated cells with Agm had no significant effect on the LPS induction of *Hif1a* (Figure 3A). In unstimulated cells HIF‐1α was undetectable, while exposure to LPS stimuli resulted in a time‐dependent increase in HIF‐1α protein levels that peaked 24 h after activation (Figures 3B and S3). Agm significantly decreased HIF‐1α levels in LPS‐exposed cells 3 and 6 h after stimulation (by 45% and 41%, respectively), displaying the most prominent effect (reduction by 68%) at the end (24 h post‐stimulation) of the experiment (Figure 3B). Immunofluorescence imaging confirmed nuclear localisation of HIF‐1α 3 h after stimulation (Figure 3C). These results were consistent with the Western blot results and suggest the ability of Agm to attenuate the LPS‐induced pseudohypoxic response by suppressing the HIF‐1α.

### 3.4 Agmatine attenuates the expression of HIF‐1α regulated genes that play a role in the glycolytic pathway

**FIGURE 4:**
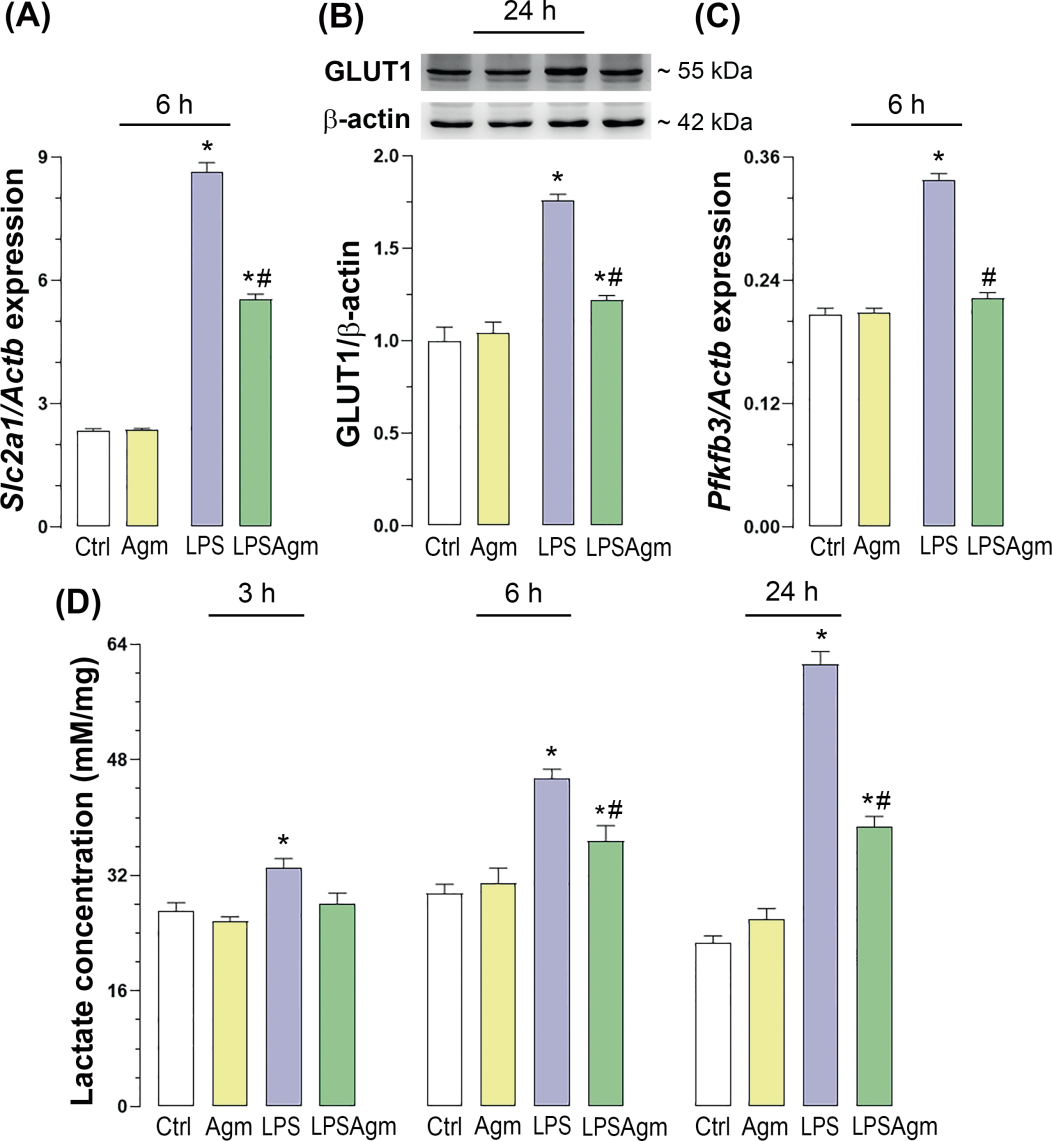
Agmatine attenuates HIF‐1α downstream responsive genes and glycolytic rate in LPS‐stimulated cells. After 3 h of starvation, BV‐2 microglia were pretreated with Agm and then stimulated with LPS for different incubation times: 6 h for qPCR analysis, 24 h for Western blot analysis and 3, 6, and 24 h for lactate determination. (A) Gene expression (*Slc2a1*) and (B) protein level of GLUT1. (C) mRNA level of *Pfkfb3*. The levels of the target genes were quantified relative to the expression of the *Actb* gene. GLUT1 protein level was normalized to β‐actin and expressed relative to the control group (fold change). (D) The extracellular lactate concentration is expressed in mM per mg proteins in the corresponding sample. All results were analyzed by two‐way ANOVA followed by Tukey's post hoc test from four independent experiments (*n* = 4) and represent means ± SEM, with the significance level determined as follows: *p < 0.05 compared to the control group; #*p* < 0.05 compared to the LPS group.

LPS had a pro‐glycolytic effect by increasing the expression of *Slc2a1* and *Pfkfb3*, genes encoding the glucose transporter GLUT1 and the enzyme PFKFB3, respectively, which are crucial for glucose uptake and regulation of glycolytic flux, while Agm significantly downregulated these genes in stimulated cells (Figure 4A,C). Agm also reduced GLUT1 protein levels upregulated by LPS (Figure 4B). Additionally, lactate production gradually increased 3, 6 and 24 h after LPS exposure indicating an increased glycolytic rate and corresponding with the protein levels of HIF‐1α (Figures 4D and S3). Agm‐treated non‐stimulated cells maintained basal lactate levels at each time point measured. Agm pretreatment of stimulated microglia significantly decreased LPS‐mediated lactate production (Figure 4D). Considering that lactate is the primary end product of glycolysis, these results suggest that LPS stimulates glycolysis via HIF‐1α, whereas Agm attenuates this effect.

### 3.5 Effect of agmatine on HIF‐1α upstream regulators

**FIGURE 5:**
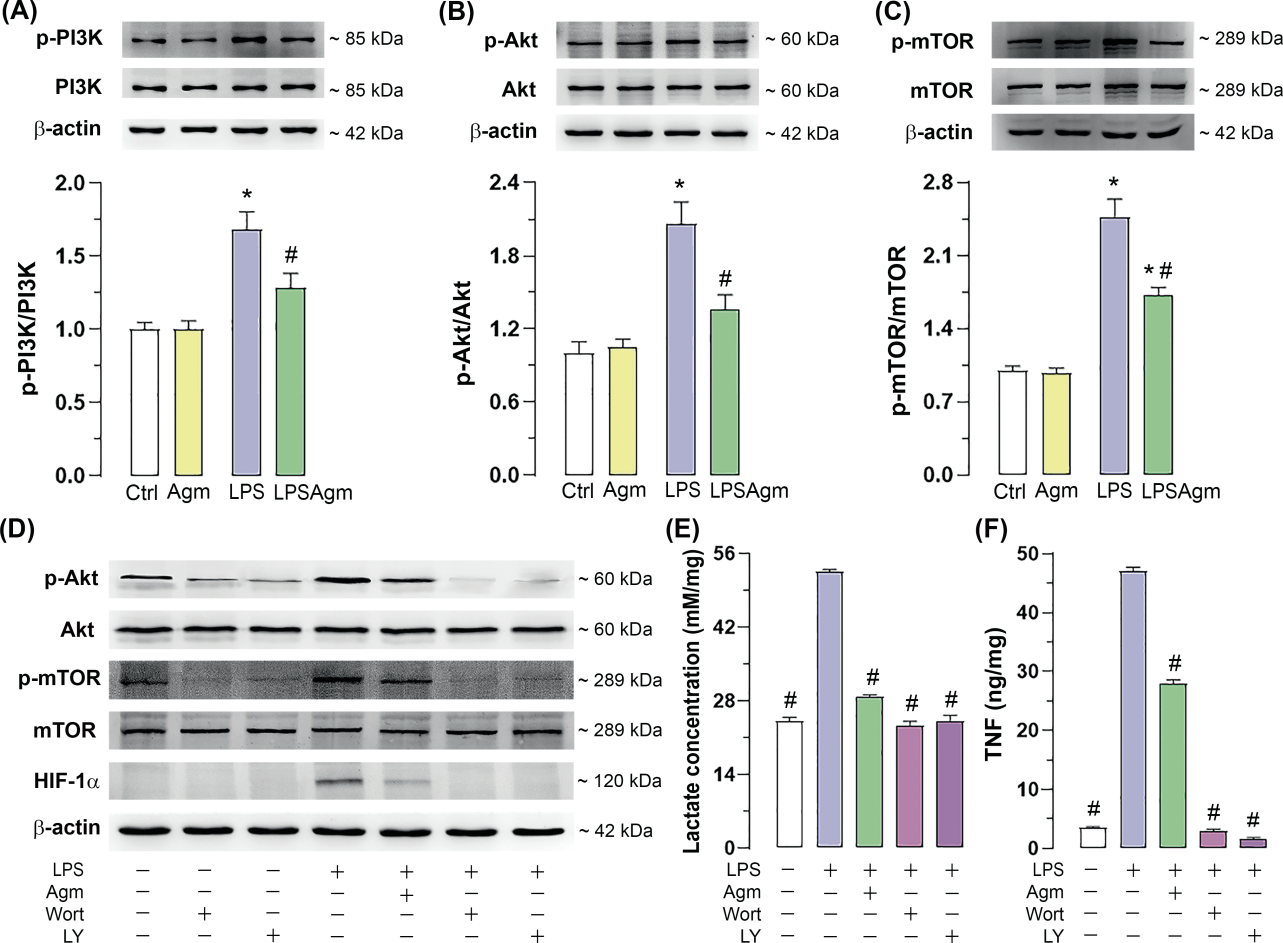
Agmatine prevents LPS‐mediated activation of the PI3K/Akt/mTOR signaling pathway in stimulated BV‐2 microglia. Cells were pretreated with 100 μM Agm for 30 min and then exposed to LPS for 15 min (for PI3K/Akt/mTOR analysis). In the case of PI3K pharmacological inhibition, cells were exposed to inhibitors wortmannin (Wort, 100 nM) or LY294002 (LY, 10 μM) for 1 h followed by 15 min (for PI3K/Akt/mTOR analysis), 3 h (for HIF‐1α analysis) or 24 h (for lactate and TNF analysis) of LPS stimulation. Agm effect on protein levels of p‐PI3K, PI3K (A), p‐Akt, Akt (B), p‐mTOR, mTOR (C), and impact of PI3K pharmacological inhibition on Akt/mTOR/HIF‐1α signaling pathway (D). Quantitative analyses were performed using four independent protein isolations (*n* = 4). Representative blots are shown with β‐actin as loading control. The graphs refer to the mean ratio of the phosphorylated form to the total target protein and are expressed relative to the control (fold change) ± SEM. The results were analyzed using a two‐way ANOVA and Tukey's post hoc test. Impact of PI3K pharmacological inhibition on lactate (E) and TNF (F) concentrations normalized to protein content and expressed as mM/mg and ng/mg, respectively, in BV‐2 supernatants 24 h after LPS stimulation. Results were analyzed using one‐way ANOVA followed by Dunnett's multiple comparisons test and presented as mean ± SEM from three independent experiments (*n* = 3). *p < 0.05 compared to the control group; #*p* < 0.05 compared to the LPS group.

Since Agm pretreatment of stimulated cells does not affect LPS‐induced transcription of HIF‐1α, but protein levels are affected in the respective groups, we analyzed the PI3K/Akt/mTOR signaling pathway known to regulate HIF‐1α translation. To follow the changes in protein levels of this pathway's components, we performed a Western blot analysis 15 min after LPS application (Figure 5A–C).

Since the total levels of PI3K, Akt, and mTOR remained unchanged upon Agm and LPS administration (Figure S4), the effect of both compounds may be attributed to changes in the phosphorylation of the target enzymes. We observed that Agm pretreatment inhibited LPS‐triggered activation of PI3K/Akt/mTOR (Figure 5A–C). Additionally, pharmacological inhibition of PI3K with wortmannin (Wort) or LY294002 (LY) blocked Akt‐dependent phosphorylation of mTOR and subsequent accumulation of HIF‐1α protein (Figure 5D), without toxic effects on BV‐2 cells (Figure S1). Furthermore, PI3K/Akt inhibition, either pharmacologically or with agmatine, significantly decreased LPS‐induced lactate production and TNF release (Figure 5E,F), indicating a dual role of this pathway in regulating glycolysis and the inflammatory response.

### 3.6 Agmatine attenuates LPS‐induced mitochondrial superoxide production, prevents depolarisation of the mitochondrial membrane and alters the Bax/Bcl‐2 ratio

**FIGURE 6:**
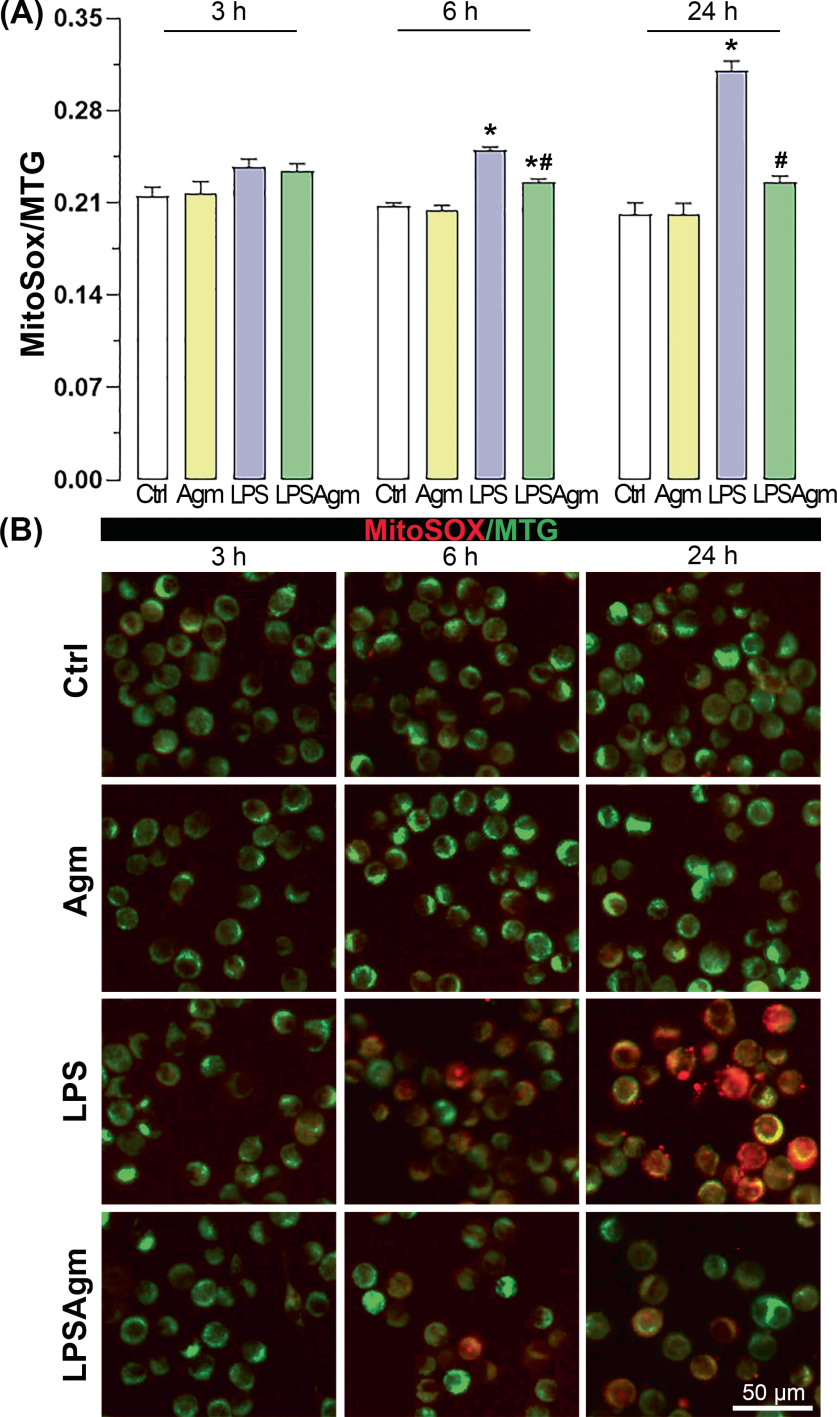
The ability of agmatine to suppress mitochondrial superoxide production in LPS‐stimulated cells. BV‐2 microglia were adapted to serum‐deprived RPMI for 3 h before being pretreated with Agm and stimulated with LPS for 3, 6, and 24 h. (A) The fluorescence intensity of MitoSOX (superoxide indicator) was normalized to the fluorescence intensity of MTG (an indicator of mitochondrial abundance). The results represent the mean of MitoSOX/MTG of four independent experiments ± SEM (*n* = 4) and were analyzed by two‐way ANOVA followed by Tukey's post hoc test with significance levels: *p < 0.05 compared to the control group; #*p* < 0.05 compared to the LPS group. (B) Representative images of BV‐2 cells incubated with MitoSOX (red fluorescence) and MTG (green fluorescence). Scale bar = 50 μm.

Mitochondrial superoxide production was measured in BV‐2 cells 3, 6, and 24 h after LPS stimulation using the fluorescent probe MitoSOX Red. LPS elicited a significant increase in mitochondrial superoxide production 6 and 24 h after stimulation, while Agm prevented this pro‐inflammatory induction of mitochondrial superoxide (Figure 6A). Additionally, after MitoSOX quantification on the microplate reader cells were subsequently visualized by fluorescence microscopy (Figure 6B).

**FIGURE 7:**
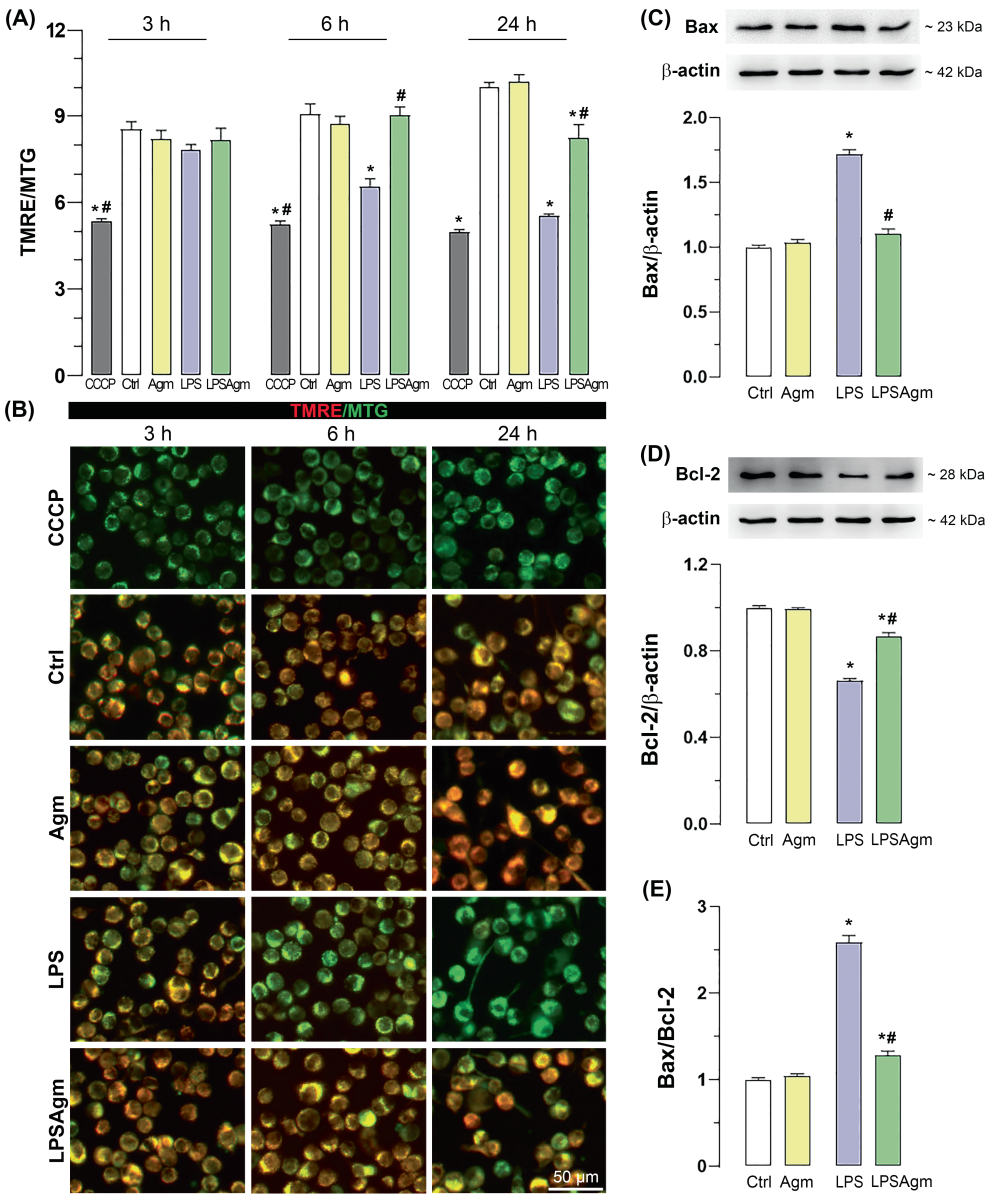
Effect of agmatine on mtMP and Bcl‐2 family proteins in control and stimulated cells. After 3 h of adaptation to a serum‐reduced medium, BV‐2 microglia were pretreated with 100 μM Agm for 30 min and then stimulated with LPS for 3, 6, and 24 h. (A) The graph shows TMRE (mtMP indicator) and MTG ratio fluorescence spectrophotometry. CCCP group served as depolarisation control. (B) The panel shows images of cells loaded with TMRE (red fluorescence) and MTG (green fluorescence). Scale bar = 50 μm. Cell lysates were collected 24 h after LPS application and analyzed by Western blotting. The graphs show the protein levels of Bax (C) and Bcl‐2 (D) relative to β‐actin as loading control and normalized to the control group (fold change). Graph (E) shows the quantified ratio of Bax to Bcl‐2. All results were analyzed using a two‐way ANOVA followed by Tukey's post hoc test and expressed as mean ± SEM from four independent experiments (*n* = 4). *p < 0.05 compared to the control group; #*p* < 0.05 compared to the LPS group.

The TMRE assay was performed to evaluate the changes in mtMP (Figure 7A). The results showed that Agm did not affect mtMP in non‐stimulated cells at any time points examined. LPS‐activation of microglia decreased the TMRE fluorescence signal 6 and 24 h after stimulation, representing membrane depolarisation, which was confirmed using CCCP‐treated cells as a depolarisation control. Application of Agm in stimulated microglia maintained mtMP at basal levels 6 h after stimulation and partially prevented its loss 24 h after stimulation (Figure 7A). After spectrophotometric measurement BV‐2 cells were visualized by fluorescence microscopy (Figure 7B).

Increased mitochondrial superoxide and mtMP are typically associated with apoptosis. Therefore, we analyzed the levels of the mitochondria‐associated regulators of apoptotic signaling, Bax and Bcl‐2, 24 h after LPS administration (Figure 7C–E). Expectedly, LPS induced an increase in pro‐apoptotic Bax (Figure 7C) and a decrease in anti‐apoptotic Bcl‐2 protein (Figure 7D), increasing their ratio by 2.6‐fold (Figure 7E). Pretreatment with Agm suppressed the LPS‐induced changes, decreasing Bax/Bcl‐2 ratio (by 50%) compared to the LPS group.

Collectively, these results show that Agm partially restores LPS‐induced mitochondrial dysfunction and prevents LPS‐triggered pro‐apoptotic signaling.

## 4 DISCUSSION

Our study discloses a novel finding that agmatine regulates energy metabolism in pro‐inflammatory activated microglia by increasing ATP yield and targeting the PI3K/Akt signaling pathway.

In homeostasis, microglia primarily rely on OXPHOS for ATP production.^2^ Our study shows that agmatine gradually increases intracellular ATP levels in unstimulated microglia, while all other analyzed markers for glycolysis or mitochondrial functionality remain unaffected. Agmatine can inhibit various nitric oxide synthase (NOS) isoforms^25^ and can enter brain cells mitochondria via a specific electrophoretic mechanism.^29^ We propose that agmatine upregulates ATP synthesis in mitochondria by inhibiting nitric oxide (NO) production from mitochondrial sources. NOS isoforms are identified in mitochondria and their role in regulating macrophage cell metabolism was recently demonstrated.^35^, ^36^ NO is thought to be a physiological regulator of mitochondrial respiration, as it can inhibit cytochrome c oxidase at low concentrations by binding to its catalytic centre.^37^ Therefore, by inhibiting mitochondrial NOS, agmatine could limit NO bioavailability in the proximity of electron transport chain and improve ATP synthesis in homeostatic microglia. The fact that agmatine can increase ATP in homeostatic microglia suggests that endogenously present agmatine may play a role in fine‐tuning of ATP production in the brain. Post‐mortem analyses have revealed that agmatine concentrations in the cerebral cortex are significantly reduced in individuals who died by suicide.^38^ Moreover, the antidepressant effect of exogenously administered agmatine is well documented in preclinical studies and a small clinical trial.^39^, ^40^ Furthermore, a lack of ATP in the prefrontal cortex, along with reduced astrocytic ATP release, has been linked with depressive behavior in animal models.^41^, ^42^ Therefore, transferring our findings on agmatine‐induced ATP production to astrocytes and animal models of depression would reinforce the importance of our results.

The administration of agmatine in the context of inflammation also led to a significant increase in ATP production over time. LPS activates inducible nitric oxide synthase (iNOS), and the microglia produce μM amounts of NO, as shown in our earlier work.^31^, ^34^, ^43^ Previous research has demonstrated that NO contributes to the OXPHOS reduction during the inflammatory response in microglia by inhibiting mitochondrial electron transport chain complexes.^44^, ^45^ Our group has shown that agmatine decreases iNOS levels and NO production in LPS‐stimulated BV‐2 cells.^31^ Thus, limited NO availability in pro‐inflammatory microglia may increase ATP production via OXPHOS, which could explain the high ATP yield observed in agmatine‐treated activated cells.

Our results show a steady increase in cellular ATP levels in LPS‐activated microglia, consistent with our previous finding.^34^ Activated immune cells are characterized by the expansion of the endoplasmic reticulum and Golgi networks to support increased production and secretion of proteins and cytokines.^46^ In line with this, our results show that LPS‐activated BV‐2 cells undergo extensive synthesis of pro‐inflammatory cytokines and cytoskeletal reorganization, significantly increasing their size compared to the homeostatic state.^31^, ^43^ These increased anabolic requirements imply an increase in ATP‐consuming systems. However, the previously reported increase in NO production in LPS‐stimulated cells^31^ may contribute to a metabolic switch to glycolysis, which is far less effective in ATP production compared to OXPHOS, resulting in a smaller increase in cellular ATP content in these cells compared to non‐stimulated cells treated with agmatine. We interpret that glycolysis is probably the main source of the increased ATP concentration in LPS‐activated cells to ensure a rapid supply of ATP, which is essential for the immediate energy demand during activation.

Our data support the role of HIF‐1α in the metabolism of LPS‐activated microglia, as we observed a gradual increase in HIF‐1α levels over time corresponding with lactate secretion. The role of HIF‐1α in the induction of glycolysis and cytokine expression has been demonstrated in the LPS‐stimulated human microglia cell line.^14^ It was also shown that the production of IL‐1 beta and TNF, together with the metabolic adaptation of OXPHOS to glycolysis, was regulated by the Akt/mTOR/HIF‐1α signaling pathway in primary microglia stimulated with either amyloid beta, LPS or ATP.^12^, ^16^ We also tracked the expression of HIF‐1α‐driven genes encoding GLUT1 (*Slc2a1*) and the PFKFB3 (*Pfkfb3*), both of which are required for the promotion of glycolysis in pro‐inflammatory polarized microglia.^47^, ^48^ Accordingly, our experiments showed increased levels of the HIF‐1α protein under inflammatory conditions and upregulated expression of its response genes *Slc2a1* and *Pfkfb3*, which are crucial for glucose uptake and glycolytic flux regulation, respectively. Although agmatine has been reported to induce HIF‐1α in neuronal cell lines in an in vitro model of Parkinson's disease (PD),^49^ our results show that pretreatment of LPS‐stimulated microglia with agmatine decreased HIF‐1α levels and efficiently reduced its downstream targets *Slc2a1* and *Pfkfb3*. The observation of reduced lactate production further strengthened the inhibitory effect of agmatine on glycolysis via HIF‐1α.

LPS is identified as a trigger of HIF‐1α transcription in BV‐2 cells^15^; our results support this finding. However, we observed that the early accumulation of HIF‐1α after LPS is not transcriptionally regulated. The agmatine‐induced reduction in HIF‐1α levels, either early or late, was also independent of transcription, as it did not affect HIF‐1α mRNA expression. These results suggest that sufficient transcript is present in the cell to respond rapidly to a hypoxic/inflammatory state and that translational and post‐translational regulations contribute to the accumulation of HIF‐1α protein in BV‐2 microglia. It is known that LPS activation of TLR4 receptors in microglia leads to increased signaling via PI3K/Akt, resulting in microglial activation and the release of inflammatory mediators.^43^, ^50^, ^51^ Given the evidence that the PI3K/Akt signaling pathway can induce HIF‐1α translation in an mTOR‐dependent manner and the importance of the mTOR/HIF‐1α pathway for metabolic adaptation of pro‐inflammatory microglia, we further investigated the involvement of these kinases in agmatine regulation of HIF‐1α.^12^, ^16^ Using pharmacological inhibition of PI3K, we confirmed that activation of the PI3K/Akt pathway in LPS‐stimulated microglia increases HIF‐1α synthesis in a rapamycin‐dependent manner. Furthermore, we observed that inhibiting the PI3K/Akt signaling pathway reduced lactate production and TNF release, demonstrating this pathway's dual role in facilitating glycolysis and inducing pro‐inflammatory effects. Similarly, agmatine regulated the phosphorylation of PI3K and its downstream targets Akt and mTOR, resulting in decreased HIF‐1α, lactate and TNF levels. Our results disagree with previous report of agmatine as a PI3K/Akt activator in the LPS‐stimulated macrophages.^52^ The discrepancy can be attributed to the timing of the PI3K/Akt analysis, which was performed relatively late (24 h) after LPS activation in the macrophage study. In support of our findings, agmatine was reported to inhibit TLR4/MyD88 signaling in LPS‐activated BV‐2 cells.^32^ Other studies on BV‐2 cells showed that activation of TLR4/MyD88 triggers PI3K phosphorylation and promotes the inflammatory response.^50^, ^51^ Therefore, agmatine likely inhibits PI3K/Akt activity by downregulating TLR4/MyD88 signaling, and this agmatine effect may be specific to the immune response of microglial cells.

Our results contrast the assertion that increased mtMP is required for the pro‐inflammatory response to LPS.^20^ We found that LPS acted as a depolarising stimulus for microglial mitochondria at two specific time points, 6 and 24 h after LPS exposure. This result aligns with other studies that report mtMP dissipation induced by LPS in microglial cells. For instance, De Simone et al. reported both mtMP‐depolarisation (1 and 24 h after LPS) and ‐hyperpolarization (6 h after LPS) in primary microglia.^19^ Similarly, disruption of mtMP towards depolarisation has been observed in LPS‐stimulated BV‐2 cells.^53^, ^54^, ^55^ LPS‐induced mtMP hyperpolarization was achieved with 10–100 ng/mL doses in primary microglia and macrophages.^10^, ^19^, ^20^ However, in the BV‐2 cell line, the depolarising effect of LPS was observed at 1 μg/mL dose or the lower dose with prolonged exposure (400 ng/mL, 48 h).^53^, ^54^, ^55^ This suggests that although BV‐2 cells and primary microglia share many similarities, there may be time‐ and dose‐dependent cell‐specific differences in the response to LPS. Regardless of the polarization state of the mitochondria, it was observed that LPS‐induced increased mitochondrial superoxide production, which is also confirmed by our results.^10^, ^11^, ^19^, ^55^ The simultaneous induction of high NO concentrations and superoxide in LPS‐stimulated microglia can promote peroxynitrite formation in mitochondria, leading to mitochondrial depolarisation by opening the permeability transition pore.^31^, ^37^, ^56^ Agmatine has been shown to protect brain mitochondria from Ca^2+^‐induced inner mitochondrial membrane permeability and mtMP loss by scavenging ROS, consequently reducing the amount of pro‐apoptotic Bcl‐2 proteins.^57^, ^58^ We have previously demonstrated that agmatine reduces cellular superoxide production in pro‐inflammatory microglia and observed the same effect on mitochondrial superoxide formation here.^31^ Therefore, the ability of agmatine to restore the mtMP is supported by agmatine's inhibition of superoxide and NO synthesis. The loss of mtMP is usually associated with apoptosis and cell death. Pro‐apoptotic marker Bax binds to the mitochondrial outer membrane and induces mitochondrial permeabilisation. We observed that an upregulation of Bax accompanied sustained depolarisation in stimulated cells and confirmed the positive effect of agmatine on mitochondrial functionality by decreasing Bax and increasing anti‐apoptotic Bcl‐2 levels.

We have uncovered two previously unknown ways in which agmatine impacts microglia. First, agmatine disrupts signaling pathways that have a dual role in triggering inflammatory responses and regulating immunometabolism. Agmatine effectively curbs glycolysis and reduces TNF in pro‐inflammatory microglia by early inhibition of PI3K/Akt/mTOR/HIF‐1α after LPS stimulation. Second, it enhances OXPHOS in homeostatic microglia, potentially aiding in the transition of pro‐inflammatory to anti‐inflammatory activation phenotype cells or inducing an anti‐inflammatory state in newly recruited microglia. Notably, agmatine enhances mitochondrial function in LPS‐activated microglia, meeting the criteria for anti‐inflammatory polarization. However, the generality of the effects of agmatine in response to other inflammatory stimuli related to sterile neuroinflammation, such as IFN or TNF, in the context of metabolic reprogramming remains to be investigated. This would be significant in estimating agmatine therapeutic potential in various inflammatory setups.

Even though the results presented here could be significant in shedding light on the benefits of agmatine, some limitations of this study need to be addressed. First, the measured concentrations of agmatine in the rat brain are estimated to be between 1.5 and 8.5 μM.^59^ Although we used a dose that is not toxic to BV‐2 cells, it well exceeds the concentrations of endogenous agmatine. Second, aside from BV‐2 cells being similar to primary microglia, they harbor oncogenes and exhibit increased proliferation, which could affect their response to agmatine treatment. However, it is noteworthy that the exact dosage of 100 μM agmatine has also proven non‐toxic to primary microglial cells.^59^ Third, our hypothesis regarding inhibiting mitochondrial NOS by agmatine in homeostatic microglia must be substantiated by detecting NO in low concentrations and distinguishing between cytoplasmic and mitochondrial NO. Finally, we would like to clarify that agmatine is probably not a classical inhibitor of PI3K, as it does not affect the phosphorylation of this protein in homeostatic cells. Since LPS–TLR4 interaction simultaneously activates the PI3K and NF‐kB signaling pathways and agmatine can suppress both (shown here and in Milosevic et al.^31^), the effects of PI3K/mTOR inhibition may be indirect and mediated by alternative mechanisms.

Although the translational relevance of our in vitro results remains to be investigated, we suggest two avenues for future research with animal models that could build on our in vitro results. The first, which we have already mentioned, is to investigate the relationship between agmatine and ATP production in animal models of depression. The second avenue is to utilize agmatine as a drug targeting HIF‐1α in the context of the role of HIF‐1α in neurodegenerative diseases. Both inducers and inhibitors of HIF‐1α have been identified as neuroprotective, depending on the target cell.^60^ For instance, silencing or inhibiting HIF‐1α in microglia was beneficial in animal models of PD and Alzheimer's disease.^14^, ^61^ Therefore, considering our finding that agmatine inhibits HIF‐1α in pro‐inflammatory microglia and the reported HIF‐1α‐inducing effect in neurons,^49^ it is plausible that agmatine may regulate HIF‐1α in a cell‐specific manner. Our study raises the intriguing question of whether the beneficial effect of agmatine demonstrated in animal models of the above‐mentioned neurodegenerative diseases^62^, ^63^ is achieved, at least in part, by differential modulation of HIF‐1α in different CNS cells. Substantiating this hypothesis in vivo would significantly enhance the therapeutic potential of agmatine as a HIF‐1α modulator.

## AUTHOR CONTRIBUTIONS

Conceptualization: Danijela Savic and Katarina Milosevic; Methodology: Katarina Milosevic; Formal analysis and investigation: Katarina Milosevic, Danijela Savic, Ana Milosevic, Anica Zivkovic; Writing – original draft preparation: Danijela Savic and Katarina Milosevic; Writing – review and editing: Ana Milosevic, Ivana Stevanovic, Anica Zivkovic, Danijela Laketa, Marija M. Janjic, Ivana Bjelobaba, Irena Lavrnja; Visualization: Danijela Savic and Katarina Milosevic. All authors read and approved the final manuscript.

## FUNDING INFORMATION

This research was supported by the Ministry of Science, Technological Development and Innovation of the Republic of Serbia (grant number 451‐03‐66/2024‐03/200007).

## CONFLICT OF INTEREST STATEMENT

The authors have no relevant financial or non‐financial interests to disclose.

## Supporting information


**Data S1.** Supporting Information.

## Data Availability

The data that support the findings of this study are available from the corresponding author upon reasonable request.
