# Supplementary material for: Agmatine suppresses glycolysis via the PI3K/Akt/mTOR/HIF‐1α signaling pathway and improves mitochondrial function in microglia exposed to lipopolysaccharide
Source: Biofactors. 2025 Jan 30;51(1):e2149. doi: 10.1002/biof.2149 (PMC11780571; doi:10.1002/biof.2149)
Supplement: Supplementary file 1 — Data S1. Supporting Information. [file BIOF-51-0-s001.pdf]

Agmatine suppresses glycolysis via the PI3K/Akt/mTOR/HIF-1α signalling pathway and improves mitochondrial function in microglia exposed to lipopolysaccharide

Katarina Milosevic[1], Ana Milosevic[1], Ivana Stevanovic[2], Anica Zivkovic[1], Danijela Laketa[3], Marija M. Janjic[1], Ivana Bjelobaba[1], Irena Lavrnja[1], Danijela Savic[1*]

[1]Department of Neurobiology, Institute for Biological Research "Sinisa Stankovic"—National Institute of Republic of Serbia, University of Belgrade, Belgrade, Serbia; katarina.tesovic@ibiss.bg.ac.rs (K.M.); ana.milosevic@ibiss.bg.ac.rs (A.M.); anica.zivkovic@ibiss.bg.ac.rs (A.Z.); marija.janjic@ibiss.bg.ac.rs (M.M.J.); ivana.bjelobaba@ibiss.bg.ac.rs (I.B.); irenam@ibiss.bg.ac.rs (I.L.); danisto@ibiss.bg.ac.rs (D.S.)

[2]Medical Faculty of the Military Medical Academy, University of Defense in Belgrade, Belgrade, Serbia; ivana.stevanovic@mod.gov.rs

[3]Department for General Physiology and Biophysics, Faculty of Biology, University of Belgrade, Belgrade, Serbia; danijela@bio.bg.ac.rs

* Corresponding author: danisto@ibiss.bg.ac.rs

Supplementary Materials:

Crystal Violet Cell Viability and MTT Assay

BV-2 microglia were seeded in a 96-well plate (20,000 cells/well). After the medium replacement procedure, cells were incubated with wortmannin (Wort) and LY294002 (LY), 1 h before LPS stimulation for 24 h.

A crystal violet assay examined whether agmatine treatment, PI3K inhibitors or LPS stimulation affected cell viability. After rinsing with PBS, cells were fixed with cold 4% PFA for 20 min at 4 °C and then incubated with 0.5% crystal violet solution (Sigma-Aldrich, Munich, Germany) for 30 min in the dark. The cells were rinsed with tap water and air-dried overnight. The next day, 33% acetic acid was added to dissolve the crystal violet dye bound to the DNA and proteins of the live cells, and the results were analysed using a microplate reader (Synergy H1M) at 570 nm absorbance.

Cellular metabolic activity was evaluated by the MTT reduction assay, following the protocol outlined by Milosevic et al. (1).

Data are expressed as a % of control mean optical density ± SEM from 5 independent experiments performed in quintuplicate per group.

Cell morphology analysis

BV-2 cells were seeded on PLL-coated Ø25 glass coverslips (50,000 cells), treated and then fixed in cold 4% PFA for 20 min at 4°C 24 h after LPS stimulation. Next, the cells were permeabilised with 0.25 % Triton X-100 for 15 min, washed in PBS and blocked with 5 % BSA for 30 min. BV-2 microglia were then incubated for 30 min in the dark at room temperature with ActinRed555 reagent (Invitrogen by Thermo Fisher Scientific, Waltham, MA, USA) containing tetramethylrhodamine-conjugated phalloidin for F-actin labelling. After washing step, the nuclei were stained for 5 min in the dark with Hoechst 33342 dye. The cells were visualised and imaged using a Zeiss Axiovert fluorescence microscope. Each cell in the micrographs was outlined using the appropriate tool in AxioVision software (version 4.9.1, Zeiss, Germany), followed by automatic measurement of the cell surface. The

analysis was performed on approximately 250 cells per group. The results are expressed as mean cell surface area (μm^2) ± SEM.

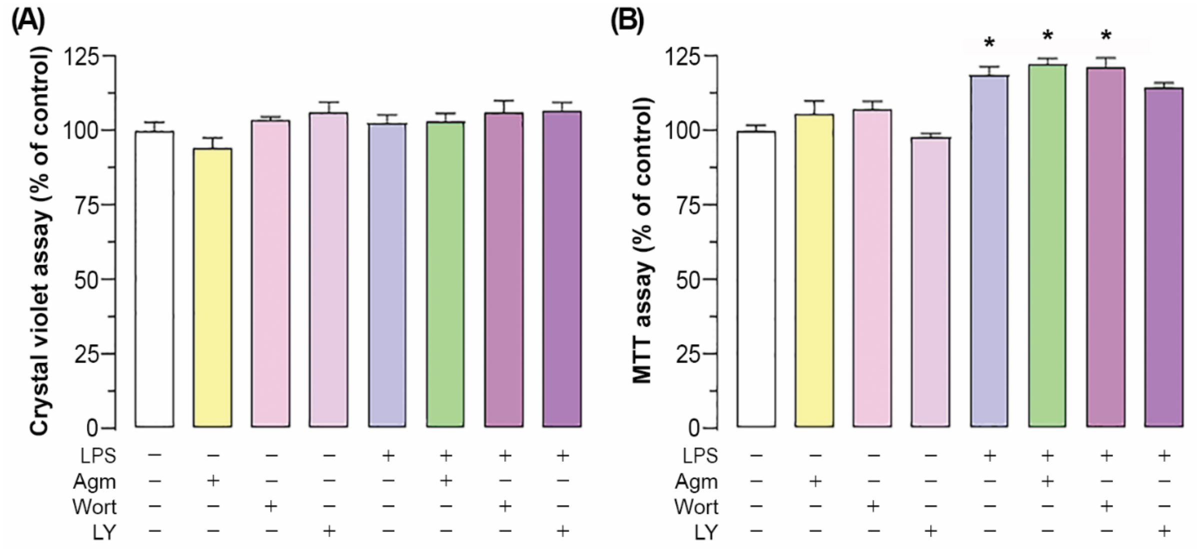

Figure S1. Effect of agmatine, LPS, and PI3K inhibitors, wortmannin (Wort) and LY294002 (LY), on BV-2 microglial cell number and metabolic activity. Cells underwent 3 h of adaptation on serum-reduced conditions before exposure to Wort (100 nM) and LY (10 μM) for 1 h or to Agm (100 μM) for 30 min. Subsequently, the cells were stimulated with LPS (1 μg/mL) for 24 h. (A) Cell number evaluated by crystal violet staining. (B) MTT colourimetric assay for measurement of cell metabolic activity. Data are presented as mean % of control ± SEM from at least five independent experiment ($n \geq 5$) and analysed by the Kruskal–Wallis test followed by Dunn's multiple comparisons test. * $p < 0.05$ compared with a control group.

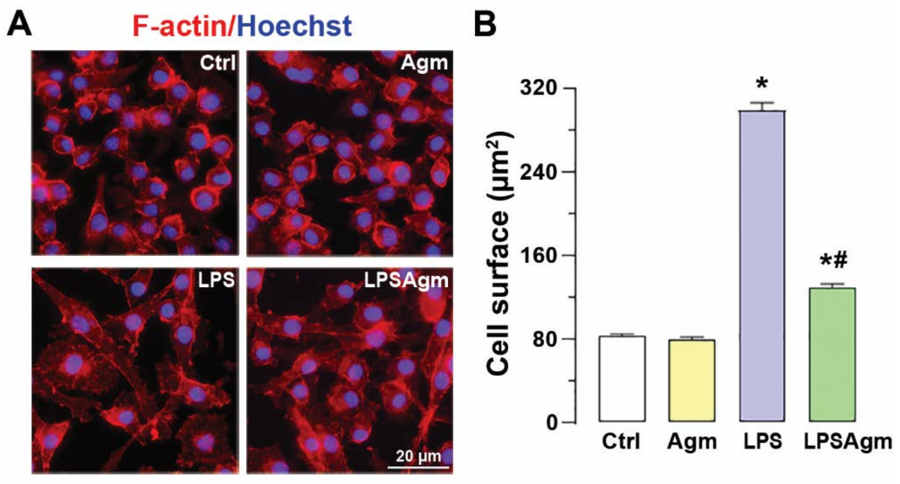

Figure S2. Effects of agmatine and LPS on the morphology of BV-2 microglia assessed by F-actin labelling. BV-2 cells underwent a 3 h assimilation process in serum-reduced medium. Afterwards, cells were pretreated with Agm (100 µM) for 30 min and stimulated with LPS (1 µg/mL) for 24 h. (A) Representative micrographs of BV-2 microglia labelled with F-actin (red) and counterstained with Hoechst (blue). Scale bar = 20 µm. (B) The graph represents the quantification of cell surface area measured in ~ 250 cells per experimental group using AxioVision software. The results were analysed using the Kruskal–Wallis test followed by Dunn's multiple comparisons test and expressed as mean cell surface area (µm^2) ± SEM. * $p < 0.05$ compared to the control group; # $p < 0.05$ compared to the LPS group.

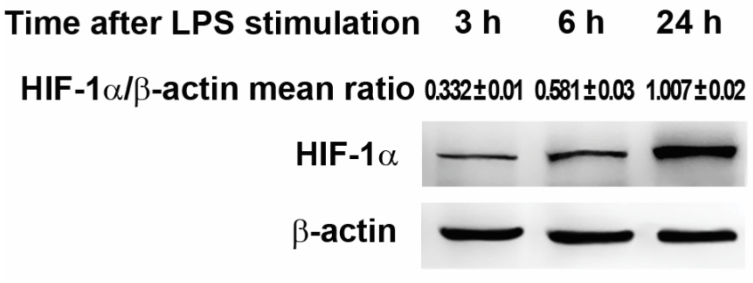

Figure S3. HIF-1α protein levels in BV-2 microglia stimulated with LPS for 3, 6, and 24 h. The representative blot shows the time-dependent upregulation of HIF-1α protein by LPS

under non-hypoxic conditions. Results were quantified from four independent protein isolations (n = 4), and the numerical value of the mean HIF-1α/β-actin ratio was assigned to the HIF-1α bands at each time point analysed. These results refer exclusively to the LPS-stimulated group.

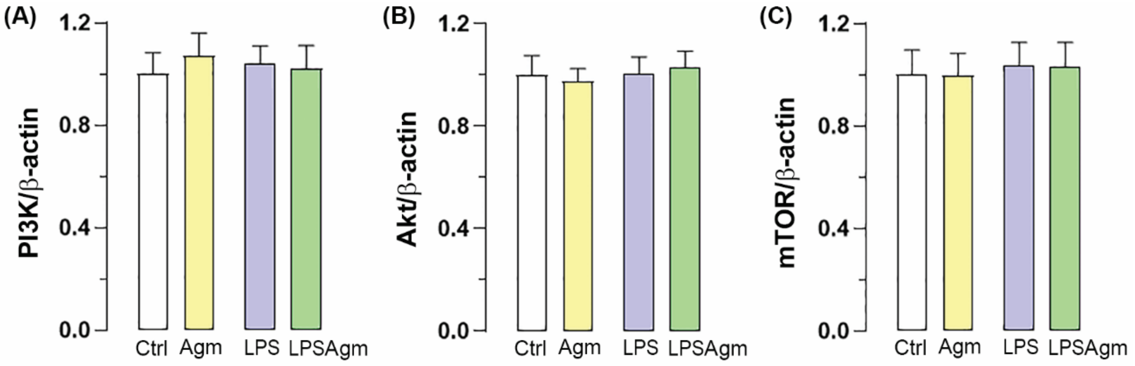

Figure S4. Agmatine treatment has no significant effect on the protein levels of total PI3K, Akt, and mTOR in non-stimulated and LPS-stimulated cells 15 min after LPS application. The graphs show total PI3K (A), Akt (B), and mTOR (C) protein levels relative to β-actin (representative blots are depicted in Figure 5). Data are presented as fold change of control ± SEM from four independent experiments (n = 4) and were analysed by two-way ANOVA followed by Tukey's post hoc test. No statistically significant difference in protein levels of total PI3K, Akt and mTOR was observed between the groups.

References:

[1] Milosevic, K., Stevanovic, I., Bozic, I.D., Milosevic, A., Janjic, M.M., et al. (2022) Agmatine Mitigates Inflammation-Related Oxidative Stress in BV-2 Cells by Inducing a Pre-Adaptive Response. *Int J Mol Sci.* **23**.
